# Supplementary material for: Molecular basis for the activation of outer dynein arms in cilia
Source: Nat Struct Mol Biol. 2025 Sep 29;32(12):2454–64. doi: 10.1038/s41594-025-01680-9 (PMC12700831; doi:10.1038/s41594-025-01680-9)
Supplement: Supplementary file 1 — Supplementary Fig. 1 containing sequences of constructs. [file 41594_2025_1680_MOESM1_ESM.pdf]

nature structural & molecular biology

Supplementary information https://doi.org/10.1038/s41594-025-01680-9

Molecular basis for the activation of outer dynein arms in cilia

In the format provided by the authors and unedited

Supplementary Figure 1

Dnaaf9-GFP sequence (only partial sequence of GFP shown)

(yellow: DNAAF9 sequence; green: part of GFP tag sequence;
red: synonymous mutation)

```
ATCCCATCGATTCGAATTCAAGGCCTCTCGAGCCATGCTTTCTAAATACCAAACAACGAACATGGCTGCTATAAG
GAAAAACAAATCTTCTATAATGACCGCTGCCGTAAGCTGCTCCCGCTTACGACAGGTCCAGGCTCTTCTAAGGGA
TGATGTCAATACAGCTCCAGATGGCATCCTTTGCTCCCTGGGTATTGACAGCCGCTACAATGAAGGCTGCAGTGA
GCTGGCCAGCTACCTTTTCTGCGGTTTGTACAACCACAACCATTTTGACATGGAGCAGATTCCAGAGGATTTCCC
TGAGGAAGTACTGGATGATGTGATCATCCTCATTAAGGCAGAGTGTGTGCATCTGTACTGCAACCCAGTGAATTA
TGGGTACCTGCTTCCCTATGTATCTCACTGGAGGAATTTACAACTTCATTGTCTGACTGAAACAGAGTACGAGGA
TGAAGAGGTTGCAGAGGAATTTAAGATCTCCAGTTTTGTGTCCATGGTTCAAGATTGCCATTGTATTGGAATCCC
CTACAGTTCTCATGGACACGTTCAGAAGTTCGATATGTTCATGTTGGAGAAATGGCCCATCATTCAGGCATTTGC
CTTGGAAGGAATTGGAGCTGGACTGTTTTTCACCATGAAATATAAGCTCAAAGATGTAAGTCAGAGACTCTGGCA
AGTCTACAGCAGATTGGACCCTGTGTCTCTGGACTGCCTTCTCAATGAGGACCTTCAGTTGTTTGAAAGACAATG
GAGCTGTTTGTTCTCCAGTATGGAAATTGAAAGTGCTCTGTCCATGCAGGAGCTGTCAGAGGCCCAAGTTGCAGA
GCCTTTCAGAACATATTATTCGCATGGTCTTATTTCCAGCAATATTACAGACAAGAGTAAAAGCAGGCAGCCATT
TGTCTTGTTTGGAAGTCACTCCACTAAGGAAGATTTGGAAAGCTATTGTTTCACCTTCCCCTCTGAAGGCCATCA
GGTCCGCAACACTGGGCCCAGCGGGGGTGTTGCCAAACACATGCTGCTTCAGTGTGTGGCTCCTAAAGGGCCACT
GGCCTGTGCTCGAACCTATTTTTTTGGAAACACACATGTCCCATACTTGGGAAATAACAACACTCAGCAGAGGAG
GACAGATCTCCAGGTGCTTTCACATATCTACTCAGCGGCAATTCAGTCTGTACTAGCAGGAATCAAATGTTTTTC
CATCAACTCTAGTGCAAGCAAGGCAAAGGATGTAGCAGATCAAACGTTTCAGCTGGCCTTGGAAAGCTTTGGGTT
GATTCAGTATAAGGGTGCTCTCAGGTCCAAAGCTGTGTTCAGCATACAGGCTGTCAACAACAAGGGGTCAATCGT
TCCCCTTAGTGATGAGGACAGCCACTTTCTGGTAAAAACAGCGTCCATGATGGTGTACGATATCCCAGATCTCCA
GTGTGGAGGAAACCTAGGATCAGTAGTTTTCTCTGAATCCTTTCTAGAGTCCAGTGTTTACATTCAGCAGAGAGA
TGGCGCTCTTTCTTCTGACAGCTGTTTCACTGTGCTCACCTCCTCAATTCCTCGCTATGTGTCCTGGCTGGTAGA
TGAAGCTGAAGTGAGAATGTCGGAACAGGCTCAACATCTGCTGAAGGCAGAGGACAGTACGTGTTTGGGCATTCC
TATAACAGTGAGAGACTCTGCCTACATGTTCTCCAACAGCCTGCTGTCCACACCTGAGGAAGGGAAGTTGGTATT
TTTCTCGGAGGGAATCTTGTTTGTGCATCCACATCATGGCTCAATAACTCTGTCTATGAGTCACATCAATAGCAT
TAAACTGTATGATGGGGCCTCTCTGTCTGATATTTCAATGCTGTTCATAGAGTATCAGACTTCTCTGCTTCCACA
CCTTCCGTTCCCATTACACAGCAGTGATTTCTCCCTGGCCGTGGCTCTGCTTCCCAGAACCAAGAGCTACAAAAG
CTTTTATTCACAGGTCCTGCCTGCTTGGCGTAAGTCAGAGTCTGGTCTGCGAGTGCAGCACGTCCTCAACGATCA
GCTGAGCCCTGAGCACAAAAGCATGTATTTCAGGCTGTTGAAGCTTCATGAGACACATGCAGTACCGGCAAACCA
CAGAGTTGTTCTGAGGACTGCTTATCCACAGCTGCCAGAGCAGGACAGATTCCTGCAGCATTTTGCAATCAGCAG
TAGTGTGGATGAAGAGTCTGTCTGTAGCGACCATTTGTCTACTGTGTTTTCAGACACAATACCTGAGAGCACCAA
ACCTGAACCCAAGAAAAAGGTGGTCCTCACTATCATTGCAGGTTTACCAGGCAGTCATAAGGAGAATCTGTGTGA
TTTCCTGATGGAAGTGAATCAGAACAGCTCCAGGTGGGAGGTGTTTTGCCCTGCTCTGGAAGGCTCTGGGGAATT
CAGCGCATCACACCTCCAGCGGTTCCTGAGCAGCCTTCTGGCCAAACAGAGAGAGACAGATATGAACTCATTCAG
AGTTGTCCTGCTTATACCAGGGTACACTGATATAATGGATGTAATCCAGGCAATCACAGCTCATCCAGACCCACA
AGTTCACAGCCAAGTGACAGTGGGTGCCGTCTCAGCTTGTGTCAACCCACTCACATCCTTCATCAAACACAGGCT
TCTCTTTCCCAAACTCCTGGAGCAGTGCAGTCAGGGCATCGTCAGCAATGTTGTCTTCACGGGTTTGACTATGGA
GCAAAAGCACCCACTATTACAACACATGCAGCAACTGATCCGCGCAGCCAATCTCAGCACTGCATTCATCTCTGC
AGAGAAAGGGGCTGTTAGAAGGACTGAAGATCTAAGACTTATTCTGAATGACAGCAGTTTCTCACAGCCTCATAT
GGTCAATGCACGATACCTGCTGTATCCTGGCTGGTGGGAGGGCAGGTTTGTGTCTGGAAGTGGATCTTTGTCTAT
GTCTCAGCATCGTGTTGAGTTCAGTCGTCCATTAGAGAGAGCATTATTTCTGCAGCGCTGCAAAGCTCTGAAATC
CTCGCTCAAACCCAGTCCTTTCACAGGAAACATCTATCACATCAGTGGGAAAGTGCTGTTTTCTGACAATGACAG
ACAGATGGTGGTAAACTGTAACTCTATCAGTGGGAATGTGACCGTTGCCCCCGATCAGGGGACTCACCATGGCCT
ATGGACAACAAACAACTGTTATCTAATGTTTCATGGAGTTGGGCTTACCCAGGAAGGGTTGAAAGACTGGCTTCG
GCATTGTGCCAAACAGAAAATTGTGAAGAAAGTCAAGAAAAATCAGAGGACGCTCACCACAGAGGAGATTAAATA
CATTCATCTAAAAAGGCACCTGGATCCTCTCCCTCCCGGTTACTTCTACAATGGCCACCACTTTGTCAGCTTCTT
TGGGGAGAAGCAAAACTTCCATCCTCTGATGGATCGATTCATCGATGAATACGTGCAGGACGCCAATAAGGAGAT
TGAGCGCTTTAACAGAGAGGTGGACCTGCAGCCACACGCTGACCTCTTCGACCCATCTAGAATGAGTAAAGGAGA
ACTTTTCACTGGAGTTGTCCCAATTCTTGTTGAATTAGATGGTGATGTAAGGCCCC
```

Translated DNAAF9 protein sequence with c-terminal GFP tag

SHRFEFKASRAMLSKYQTTNMAAIRKNKSSIMTAAVSCSRLRQVQALLRDDVNTAPDGILCSLGIDSR
YNEGCSELASYLFCGLYNHNHFDMEQIPEDFPEEVLDDVIILIKAECVHLYCNPVNYGYLLPYVSHWR
NLQLHCLTETEYEDEEVAEEFKISSFVSMVQDCHCIGIPYSSHGHVQKFDMFMLEKWPIIQAFALEGI
GAGLFFTMKYKLKDVSQRLWQVYSRLDPVSLDCLLNEDLQLFERQWSCLFSSMEIESALSMQELSEAQ
VAEPFRTYYSHGLISSNITDKSKSRQPFVLFGSHSTKEDLESYCFTFPSEGHQVRNTGPSGGVAKHML
LQCVAPKGPLACARTYFFGNTHVPYLGNNNTQQRRTDLQVLSHIYSAAIQSVLAGIKCFSINSSASKA
KDVADQTFQLALESFGLIQYKGALRSKAVFSIQAVNNKGSIVPLSDEDSHFLVKTASMMVYDIPDLQC
GGNLGSVVFSESFLESSVYIQQRDGALSSDSCFTVLTSSIPRYVSWLVDEAEVRMSEQAQHLLKAEDS
TCLGIPITVRDSAYMFSNSLLSTPEEGKLVFFSEGILFVHPHHGSITLSMSHINSIKLYDGASLSDIS
MLFIEYQTSLLPHLPFPLHSSDFSLAVALLPRTKSYKSFYSQVLPAWRKSESGLRVQHVLNDQLSPEH
KSMYFRLLKLHETHAVPANHRVVLRTAYPQLPEQDRFLQHFAISSSVDEESVCSDHLSTVFSDTIPES
TKPEPKKKVVLTIIAGLPGSHKENLCDFLMEVNQNSSRWEVFCPALEGSGEFSASHLQRFLSSLLAKQ
RETDMNSFRVVLLIPGYTDIMDVIQAITAHPDPQVHSQVTVGAVSACVNPLTSFIKHRLLFPKLLEQC
SQGIVSNVVFTGLTMEQKHPLLQHMQQLIRAANLSTAFISAEKGAVRRTEDLRLILNDSSFSQPHMVN
ARYLLYPGWWEGRFVSGSGSLSMSQHRVEFSRPLERALFLQRCKALKSSLKPSPFTGNIYHISGKVLF
SDNDRQMVVNCNSISGNVTVAPDQGTHHGLWTTNNCYLMFHGVGLTQEGLKDWLRHCAKQKIVKKVKK
NQRTLTTEEIKYIHLKRHLDPLPPGYFYNGHHFVSFFGEKQNFHPLMDRFIDEYVQDANKEIERFNRE
VDLQPHADLFDPSRMSKGELFTGVVPILVELDGDVRP

Dnaaf9-myc sequence (5xmyc tag, only partial sequence of myc tag shown)

(yellow: DNAAF9 sequence; light blue: part of myc tag sequence; red: synonymous mutation)

GAGCTCTGGTTCTTTTTGCAGGATCCCATCGAACCATGCTTTCTAAATACCAAACAACGAACATGGCTGCTATAA
GGAAAAACAAATCTTCTATAATGACCGCTGCCGTAAGCTGCTCCCGCTTACGACAGGTCCAGGCTCTTCTAAGGG
ATGATGTCAATACAGCTCCAGATGGCATCCTTTGCTCCCTGGGTATTGACAGCCGCTACAATGAAGGCTGCAGTG
AGCTGGCCAGCTACCTTTTCTGCGGTTTGTACAACCACAACCATTTTGACATGGAGCAGATTCCAGAGGATTTCC
CTGAGGAAGTACTGGATGATGTGATCATCCTCATTAAGGCAGAGTGTGTGCATCTGTACTGCAACCCAGTGAATT
ATGGGTACCTGCTTCCCTATGTATCTCACTGGAGGAATTTACAACTTCATTGTCTGACTGAAACAGAGTACGAGG
ATGAAGAGGTTGCAGAGGAATTTAAGATCTCCAGTTTTGTGTCCATGGTTCAAGATTGCCATTGTATTGGAATCC
CCTACAGTTCTCATGGACACGTTCAGAAGTTCGATATGTTCATGTTGGAGAAATGGCCCATCATTCAGGCATTTG
CCTTGGAAGGAATTGGAGCTGGACTGTTTTTCACCATGAAATATAAGCTCAAAGATGTAAGTCAGAGACTCTGGC
AAGTCTACAGCAGATTGGACCCTGTGTCTCTGGACTGCCTTCTCAATGAGGACCTTCAGTTGTTTGAAAGACAAT
GGAGCTGTTTGTTCTCCAGTATGGAAATTGAAAGTGCTCTGTCCATGCAGGAGCTGTCAGAGGCCCAAGTTGCAG
AGCCTTTCAGAACATATTATTCGCATGGTCTTATTTCCAGCAATATTACAGACAAGAGTAAAAGCAGGCAGCCAT
TTGTCTTGTTTGGAAGTCACTCCACTAAGGAAGATTTGGAAAGCTATTGTTTCACCTTCCCCTCTGAAGGCCATC
AGGTCCGCAACACTGGGCCCAGCGGGGGTGTTGCCAAACACATGCTGCTTCAGTGTGTGGCTCCTAAAGGGCCAC
TGGCCTGTGCTCGAACCTATTTTTTTGGAAACACACATGTCCCATACTTGGGAAATAACAACACTCAGCAGAGGA
GGACAGATCTCCAGGTGCTTTCACATATCTACTCAGCGGCAATTCAGTCTGTACTAGCAGGAATCAAATGTTTTT
CCATCAACTCTAGTGCAAGCAAGGCAAAGGATGTAGCAGATCAAACGTTTCAGCTGGCCTTGGAAAGCTTTGGGT
TGATTCAGTATAAGGGTGCTCTCAGGTCCAAAGCTGTGTTCAGCATACAGGCTGTCAACAACAAGGGGTCAATCG
TTCCCCTTAGTGATGAGGACAGCCACTTTCTGGTAAAAACAGCGTCCATGATGGTGTACGATATCCCAGATCTCC
AGTGTGGAGGAAACCTAGGATCAGTAGTTTTCTCTGAATCCTTTCTAGAGTCCAGTGTTTACATTCAGCAGAGAG
ATGGCGCTCTTTCTTCTGACAGCTGTTTCACTGTGCTCACCTCCTCAATTCCTCGCTATGTGTCCTGGCTGGTAG
ATGAAGCTGAAGTGAGAATGTCGGAACAGGCTCAACATCTGCTGAAGGCAGAGGACAGTACGTGTTTGGGCATTC
CTATAACAGTGAGAGACTCTGCCTACATGTTCTCCAACAGCCTGCTGTCCACACCTGAGGAAGGGAAGTTGGTAT
TTTTCTCGGAGGGAATCTTGTTTGTGCATCCACATCATGGCTCAATAACTCTGTCTATGAGTCACATCAATAGCA
TTAAACTGTATGATGGGGCCTCTCTGTCTGATATTTCAATGCTGTTCATAGAGTATCAGACTTCTCTGCTTCCAC
ACCTTCCGTTCCCATTACACAGCAGTGATTTCTCCCTGGCCGTGGCTCTGCTTCCCAGAACCAAGAGCTACAAAA
GCTTTTATTCACAGGTCCTGCCTGCTTGGCGTAAGTCAGAGTCTGGTCTGCGAGTGCAGCACGTCCTCAACGATC
AGCTGAGCCCTGAGCACAAAAGCATGTATTTCAGGCTGTTGAAGCTTCATGAGACACATGCAGTACCGGCAAACC
ACAGAGTTGTTCTGAGGACTGCTTATCCACAGCTGCCAGAGCAGGACAGATTCCTGCAGCATTTTGCAATCAGCA
GTAGTGTGGATGAAGAGTCTGTCTGTAGCGACCATTTGTCTACTGTGTTTTCAGACACAATACCTGAGAGCACCA
AACCTGAACCCAAGAAAAAGGTGGTCCTCACTATCATTGCAGGTTTACCAGGCAGTCATAAGGAGAATCTGTGTG
ATTTCCTGATGGAAGTGAATCAGAACAGCTCCAGGTGGGAGGTGTTTTGCCCTGCTCTGGAAGGCTCTGGGGAAT
TCAGCGCATCACACCTCCAGCGGTTCCTGAGCAGCCTTCTGGCCAAACAGAGAGAGACAGATATGAACTCATTCA
GAGTTGTCCTGCTTATACCAGGGTACACTGATATAATGGATGTAATCCAGGCAATCACAGCTCATCCAGACCCAC
AAGTTCACAGCCAAGTGACAGTGGGTGCCGTCTCAGCTTGTGTCAACCCACTCACATCCTTCATCAAACACAGGC
TTCTCTTTCCCAAACTCCTGGAGCAGTGCAGTCAGGGCATCGTCAGCAATGTTGTCTTCACGGGTTTGACTATGG
AGCAAAAGCACCCACTATTACAACACATGCAGCAACTGATCCGCGCAGCCAATCTCAGCACTGCATTCATCTCTG
CAGAGAAAGGGGCTGTTAGAAGGACTGAAGATCTAAGACTTATTCTGAATGACAGCAGTTTCTCACAGCCTCATA
TGGTCAATGCACGATACCTGCTGTATCCTGGCTGGTGGGAGGGCAGGTTTGTGTCTGGAAGTGGATCTTTGTCTA
TGTCTCAGCATCGTGTTGAGTTCAGTCGTCCATTAGAGAGAGCATTATTTCTGCAGCGCTGCAAAGCTCTGAAAT
CCTCGCTCAAACCCAGTCCTTTCACAGGAAACATCTATCACATCAGTGGGAAAGTGCTGTTTTCTGACAATGACA
GACAGATGGTGGTAAACTGTAACTCTATCAGTGGGAATGTGACCGTTGCCCCCGATCAGGGGACTCACCATGGCC
TATGGACAACAAACAACTGTTATCTAATGTTTCATGGAGTTGGGCTTACCCAGGAAGGGTTGAAAGACTGGCTTC
GGCATTGTGCCAAACAGAAAATTGTGAAGAAAGTCAAGAAAAATCAGAGGACGCTCACCACAGAGGAGATTAAAT
ACATTCATCTAAAAAGGCACCTGGATCCTCTCCCTCCCGGTTACTTCTACAATGGCCACCACTTTGTCAGCTTCT
TTGGGGAGAAGCAAAACTTCCATCCTCTGATGGATCGATTCATCGATGAATACGTGCAGGACGCCAATAAGGAGA
TTGAGCGCTTTAACAGAGAGGTGGACCTGCAGCCACACGCTGACCTCTTCGACCCACATCGATTTAAAGCTATGG
AGCAAAAGCTCATTTCTGAAGAGGACTTGAATGAAATGGAGCAAAAGCTCATTTCTGAAGAGGACTTGAATGAA

Translated DNAAF9 protein sequence with c-terminal 5xmyc tag

ALVLFAGSHRTMLSKYQTTNMAAIRKNKSSIMTAAVSCSRLRQVQALLRDDVNTAPDGILCSLGIDSR
YNEGCSELASYLFCGLYNHNHFDMEQIPEDFPEEVLDDVIILIKAECVHLYCNPVNYGYLLPYVSHWR
NLQLHCLTETEYEDEEVAEEFKISSFVSMVQDCHCIGIPYSSHGHVQKFDMFMLEKWPIIQAFALEGI
GAGLFFTMKYKLKDVSQRLWQVYSRLDPVSLDCLLNEDLQLFERQWSCLFSSMEIESALSMQELSEAQ
VAEPFRTYYSHGLISSNITDKSKSRQPFVLFGSHSTKEDLESYCFTFPSEGHQVRNTGPSGGVAKHML
LQCVAPKGPLACARTYFFGNTHVPYLGNNNTQQRRTDLQVLSHIYSAAIQSVLAGIKCFSINSSASKA
KDVADQTFQLALESFGLIQYKGALRSKAVFSIQAVNNKGSIVPLSDEDSHFLVKTASMMVYDIPDLQC
GGNLGSVVFSESFLESSVYIQQRDGALSSDSCFTVLTSSIPRYVSWLVDEAEVRMSEQAQHLLKAEDS
TCLGIPITVRDSAYMFSNSLLSTPEEGKLVFFSEGILFVHPHHGSITLSMSHINSIKLYDGASLSDIS
MLFIEYQTSLLPHLPFPLHSSDFSLAVALLPRTKSYKSFYSQVLPAWRKSESGLRVQHVLNDQLSPEH
KSMYFRLLKLHETHAVPANHRVVLRTAYPQLPEQDRFLQHFAISSSVDEESVCSDHLSTVFSDTIPES
TKPEPKKKVVLTIIAGLPGSHKENLCDFLMEVNQNSSRWEVFCPALEGSGEFSASHLQRFLSSLLAKQ
RETDMNSFRVVLLIPGYTDIMDVIQAITAHPDPQVHSQVTVGAVSACVNPLTSFIKHRLLFPKLLEQC
SQGIVSNVVFTGLTMEQKHPLLQHMQQLIRAANLSTAFISAEKGAVRRTEDLRLILNDSSFSQPHMVN
ARYLLYPGWWEGRFVSGSGSLSMSQHRVEFSRPLERALFLQRCKALKSSLKPSPFTGNIYHISGKVLF
SDNDRQMVVNCNSISGNVTVAPDQGTHHGLWTTNNCYLMFHGVGLTQEGLKDWLRHCAKQKIVKKVKK
NQRTLTTEEIKYIHLKRHLDPLPPGYFYNGHHFVSFFGEKQNFHPLMDRFIDEYVQDANKEIERFNRE
VDLQPHADLFDPHRFKAMEQKLISEEDLNEMEQKLISEEDLNE
