# Supplementary material for: Molecular basis for the activation of outer dynein arms in cilia
Source: Nat Struct Mol Biol. 2025 Sep 29;32(12):2454–64. doi: 10.1038/s41594-025-01680-9 (PMC12700831; doi:10.1038/s41594-025-01680-9)
Supplement: Supplementary file 2 — Reporting Summary [file 41594_2025_1680_MOESM2_ESM.pdf]

Corresponding author(s): Girish Ram Mali

Last updated by author(s): Aug 14, 2025

Reporting Summary

Nature Portfolio wishes to improve the reproducibility of the work that we publish. This form provides structure for consistency and transparency in reporting. For further information on Nature Portfolio policies, see our Editorial Policies and the Editorial Policy Checklist.

Statistics

For all statistical analyses, confirm that the following items are present in the figure legend, table legend, main text, or Methods section.

n/a	Confirmed	
☐	☒	The exact sample size (*n*) for each experimental group/condition, given as a discrete number and unit of measurement
☐	☒	A statement on whether measurements were taken from distinct samples or whether the same sample was measured repeatedly
☐	☒	The statistical test(s) used AND whether they are one- or two-sided<br>*Only common tests should be described solely by name; describe more complex techniques in the Methods section.*
☒	☐	A description of all covariates tested
☐	☒	A description of any assumptions or corrections, such as tests of normality and adjustment for multiple comparisons
☐	☒	A full description of the statistical parameters including central tendency (e.g. means) or other basic estimates (e.g. regression coefficient) AND variation (e.g. standard deviation) or associated estimates of uncertainty (e.g. confidence intervals)
☐	☒	For null hypothesis testing, the test statistic (e.g. *F*, *t*, *r*) with confidence intervals, effect sizes, degrees of freedom and *P* value noted<br>*Give P values as exact values whenever suitable.*
☒	☐	For Bayesian analysis, information on the choice of priors and Markov chain Monte Carlo settings
☒	☐	For hierarchical and complex designs, identification of the appropriate level for tests and full reporting of outcomes
☒	☐	Estimates of effect sizes (e.g. Cohen's *d*, Pearson's *r*), indicating how they were calculated

Our web collection on statistics for biologists contains articles on many of the points above.

Software and code

Policy information about availability of computer code

Data collection	Leica SP8 Confocal Laser Scanning Microscope, Olympus FV3000 upright confocal microscope, Thermo iBright FL1000 Imaging System, FEI EPU on an FEI 200kV Tecnai 20 TEM, JEOL 2100 Plus 200kV TEM.
Data analysis	Main software or servers used for data analyses are publicly available, detailed in the manuscript methods section and included below: Fiji v2.1.0/1.53c, GraphPad Prism v10, RELION v3.2 or v4.0, UCSF ChimeraX v1.7-v1.9, Google Colab Alphafold2 Notebook (https://colab.research.google.com/github/sokrypton/ColabFold/blob/main/AlphaFold2.ipynb) and Alphafold 3 (AlphaFold web server: https://alphafoldserver.com/), PSI-BLAST, Clustal Omega (integrated in Uniprot: https://www.uniprot.org/), ESPript 3 (https://espript.ibcp.fr/ESPript/ESPript/), CONSURF server (https://consurf.tau.ac.il/consurf_index.php).

For manuscripts utilizing custom algorithms or software that are central to the research but not yet described in published literature, software must be made available to editors and reviewers. We strongly encourage code deposition in a community repository (e.g. GitHub). See the Nature Portfolio guidelines for submitting code & software for further information.

Data

Policy information about availability of data

All manuscripts must include a data availability statement. This statement should provide the following information, where applicable:

- Accession codes, unique identifiers, or web links for publicly available datasets
- A description of any restrictions on data availability
- For clinical datasets or third party data, please ensure that the statement adheres to our policy

Mass spectrometry data have been deposited in ProteomeXchange and made available via the PRIDE48 partner repository with the primary accession code PXD052722. Source data have been provided in Source Data and also available at https://doi.org/10.6084/m9.figshare.27195438. All other data supporting the findings of this study are available from the corresponding author upon reasonable request.

Research involving human participants, their data, or biological material

Policy information about studies with human participants or human data. See also policy information about sex, gender (identity/presentation), and sexual orientation and race, ethnicity and racism.

Reporting on sex and gender	N/A
Reporting on race, ethnicity, or other socially relevant groupings	N/A
Population characteristics	N/A
Recruitment	N/A
Ethics oversight	N/A

Note that full information on the approval of the study protocol must also be provided in the manuscript.

Field-specific reporting

Please select the one below that is the best fit for your research. If you are not sure, read the appropriate sections before making your selection.

☒ Life sciences ☐ Behavioural & social sciences ☐ Ecological, evolutionary & environmental sciences

For a reference copy of the document with all sections, see nature.com/documents/nr-reporting-summary-flat.pdf

Life sciences study design

All studies must disclose on these points even when the disclosure is negative.

Sample size

Human airway epithelial cells (HAECs) were obtained as transwell cultures containing separate pooled batches of cells from Epithelix Sarl at two differentiation time-points. Cell clumps were gently scraped from transwell inserts and suspended down to single cells which were then applied to glass slides and allowed to air-dry. Each slide contained several hundred individual cells which were processed for immunostainings. Many cells were observed under a confocal microscope. Of these 10-20 representative cell images/batch were randomly acquired for quantitative analyses.

All zebrafish localization experiments were performed in at least 2 biological replicates and the numbers of control and injected embryos imaged were n>4.

Sample sizes were not pre-determined for the structural analyses performed in this study. Negative stain EM data was collected until a sufficiently high resolution to answer our biological question was achieved.

Data exclusions

No data were excluded from the cell immunostaining analyses. EM micrographs that contained empty areas and thick stain in the field of view were excluded. During 2D and 3D classification, particles that did not yield detailed images to reveal structural densities were also excluded.

Replication

Immunostainings performed on three replicate batches showed similar sub-cellular distributions for endogenous DNAAF9 and DNAI2 in the immature and more mature human airway cells.

IP-MS experiments were performed in triplicate followed by 12-plex TMT mass spectrometry runs.

ODA-Shulin biochemical reconstitutions and displacement experiments using Arl3 Q70L or Arl3 Q70L-FYY were performed in triplicate and these replicate data were used for gel densitometry based quantification analyses.

Imaging of n>4 control and injected embryos from 2 biological replicates showed similar sub-cellular distribution for the C-terminal tagged versions of Dnaaf9 protein.

No attempt was made to replicate the negative stain EM data which involves averaging of several thousands of individual protein molecules.

Randomization	Cell images from randomly selected areas were acquired to allow for unbiased random sampling. ~10-20 cell images from each batch were acquired to provide data from three biologically different batches. Randomization was not relevant to the structural methods of this study.
Blinding	Three study authors (BB, MR and GRM) were involved in acquiring cell images independently. It was difficult to perform blinding as the cell slides were labeled and additional blinding was not attempted.

Reporting for specific materials, systems and methods

We require information from authors about some types of materials, experimental systems and methods used in many studies. Here, indicate whether each material, system or method listed is relevant to your study. If you are not sure if a list item applies to your research, read the appropriate section before selecting a response.

Materials & experimental systems

n/a	Involved in the study	
☐	☒	Antibodies
☐	☒	Eukaryotic cell lines
☒	☐	Palaeontology and archaeology
☐	☒	Animals and other organisms
☒	☐	Clinical data
☒	☐	Dual use research of concern
☒	☐	Plants

Methods

n/a	Involved in the study	
☒	☐	ChIP-seq
☒	☐	Flow cytometry
☒	☐	MRI-based neuroimaging

Antibodies

Antibodies used	Primary antibodies commercial: DNAAF9 (Proteintech, 23184-1-AP) DNAI2 (Abnova, H00064446-M01, clone IC8), Acetylated alpha tubulin (Cell Signaling, 5335), IFT74 (Proteintech, 27334-1-AP), IFT81 (Proteintech, 11744-1-AP), anti-Strep tag (GT661, Thermo Scientific, MA5-17283), Myc-tag (Santa Cruz, sc-40), GFP (Abcam, ab13970).<br><br>Primary antibodies custom generated: Tetrahymena ODA holocomplex (Eurogentec).<br><br>Secondary antibodies commercial: Alexa Fluor 568 anti-rabbit antibody (Invitrogen, A-11011), Alexa Fluor 488 anti-mouse antibody (Invitrogen, A-10680), Alexa 555 anti-mouse antibody (Invitrogen, A-28180), Alexa 488 anti-rabbit antibody (Invitrogen, A-11034). GeneTex, EasyBlot anti-rabbit IgG and anti-mouse IgG HRP conjugated monoclonal antibodies; GTX221666-01-S and GTX221667-01-S respectively).
Validation	Commercially available antibodies have validation and relevant citation data on the manufacturers website. DNAAF9 antibody was additionally validated in house by immunoblotting for purified recombinant DNAAF9 protein and by immunoprecipitation and mass spectrometric detection of target protein i.e. DNAAF9 as the top hit. DNAI2 antibody was previously tested in Diggle et al., DOI: 10.1371/journal.pgen.1004577.<br><br>Custom antibody raised to the Tetrahymena ODA holocomplex was previously tested and used in Mali et al., 2021; DOI: 10.1126/science.abe0526.

Eukaryotic cell lines

Policy information about cell lines and Sex and Gender in Research

Cell line source(s)	Sf9 cells were obtained from the Eukaryotic Expression Facility (EEF) operated by the Berger lab at the University of Bristol's School of Biochemistry.<br><br>Human airway epithelial cells (HAECs) were obtained as transwell cultures containing separate pooled batches of cells from multiple donors from Epithelix Sarl (Switzerland).
Authentication	None of the cell lines were authenticated by the study authors. All cell lines or primary cells were obtained from reliable sources with in-house quality control procedures in place.
Mycoplasma contamination	Cell lines tested negative for mycoplasma contamination.
Commonly misidentified lines (See ICLAC register)	N/A

Animals and other research organisms

Policy information about studies involving animals; ARRIVE guidelines recommended for reporting animal research, and Sex and Gender in Research

Laboratory animals	Mice, Zebrafish
Wild animals	Study did not involve wild animals.
Reporting on sex	Both male and female animals were equally analysed. 8 male and 8 female adult mutant animals were phenotyped. Brain histopathology was performed on 2 male and 2 female homozygote adult animals. Motile cilia are fundamental to the physiology of multiciliated cells and it is unlikely that the findings would apply specifically to only one sex.
Field-collected samples	Study did not involve samples collected from the field.
Ethics oversight	Mice - All procedures on animals at The Centre for Phenogenomics (TCP) were reviewed and approved by TCP's Animal Care Committee. TCP is certified by the Canadian Council on Animal Care and registered under the Animals for Research Act of Ontario. TCP is part of the International Mouse Phenotyping Consortium.<br><br>Zebrafish - All experiments with zebrafish embryos were approved by the Singapore National Advisory on Laboratory Animal Research.

Note that full information on the approval of the study protocol must also be provided in the manuscript.

Plants

Seed stocks	N/A
Novel plant genotypes	N/A
Authentication	N/A
