# Supplementary material for: Molecular basis for the activation of outer dynein arms in cilia
Source: Nat Struct Mol Biol. 2025 Sep 29;32(12):2454–64. doi: 10.1038/s41594-025-01680-9 (PMC12700831; doi:10.1038/s41594-025-01680-9)

Gel and western blot source data, Figure 2

Figure 2c

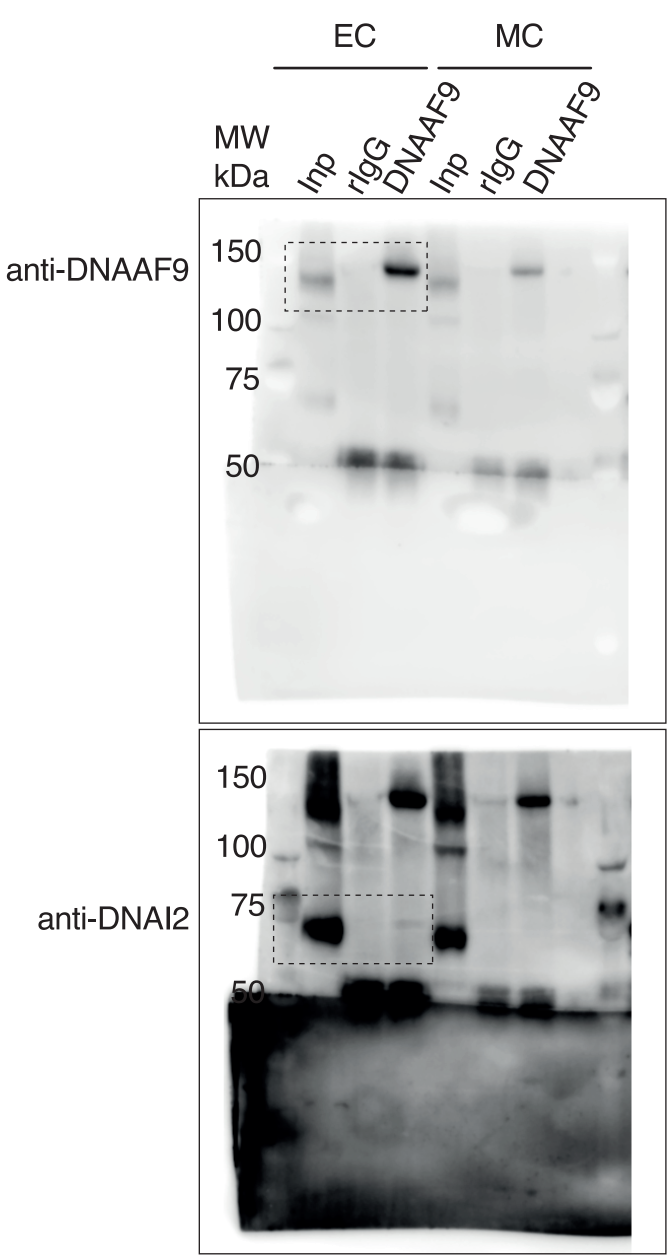

Figure 2g

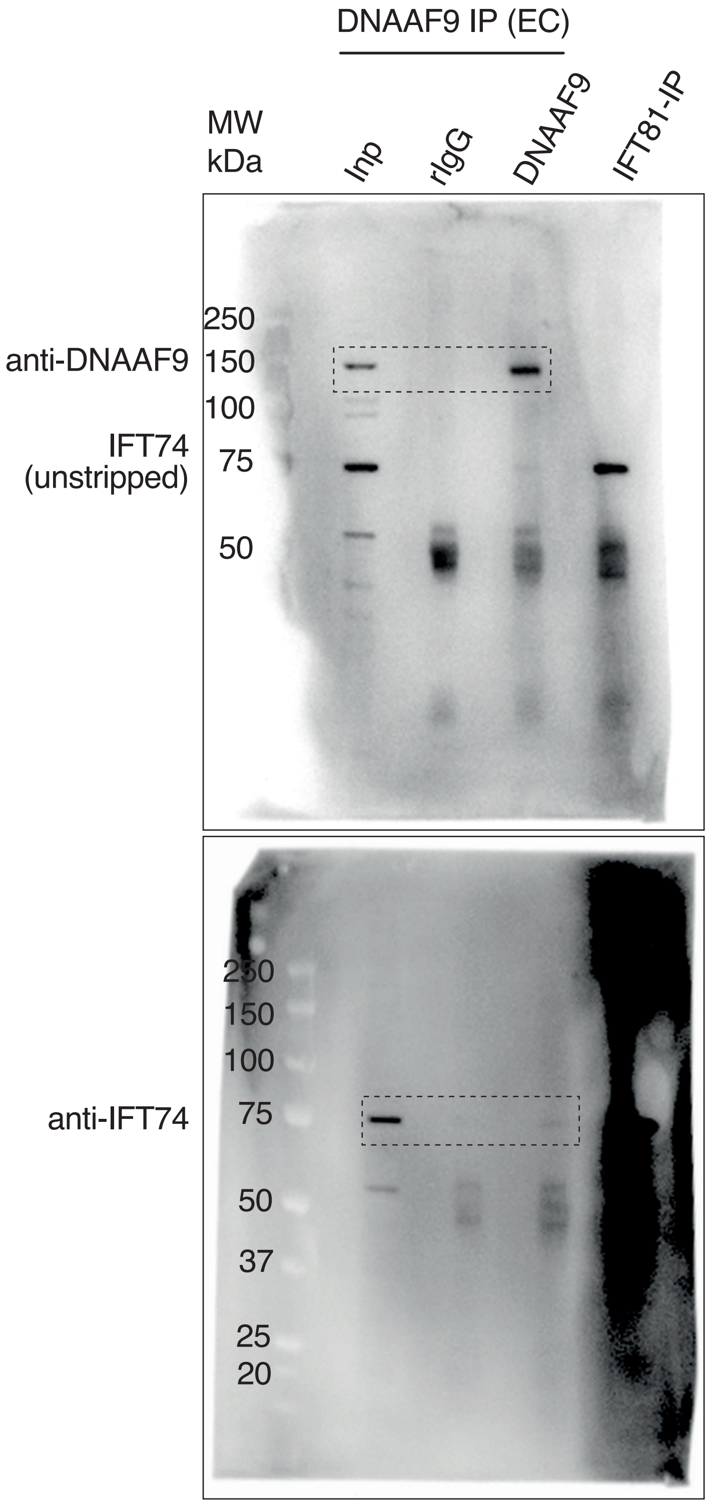

Figure 2h

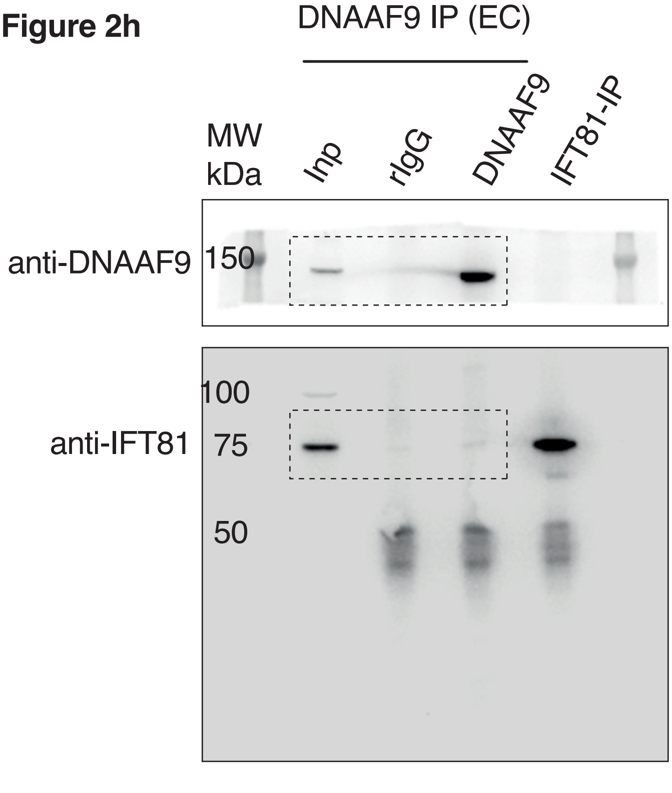

Regions shown in figures 2c, 2g and 2h are indicated by dashed boxes

Gel and western blot source data, Figure 3

Figure 3b

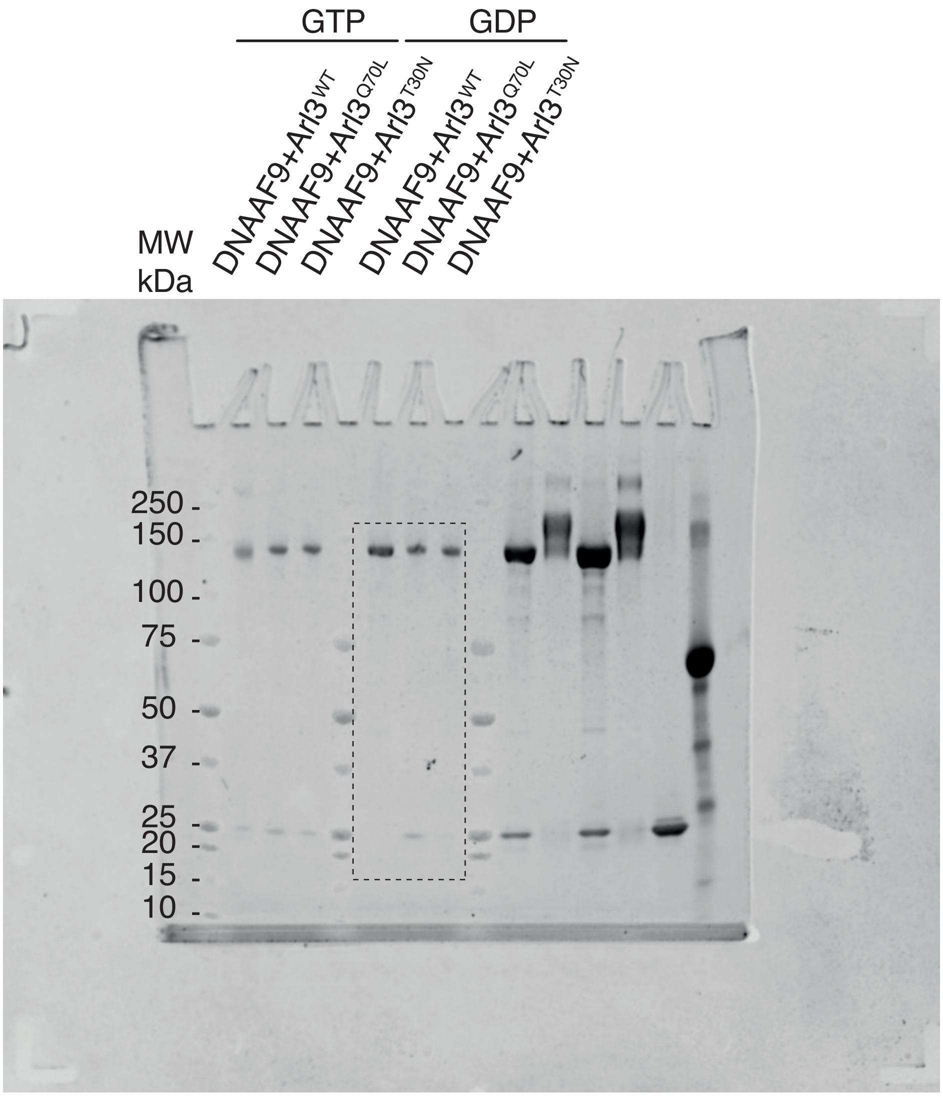

Figure 3d

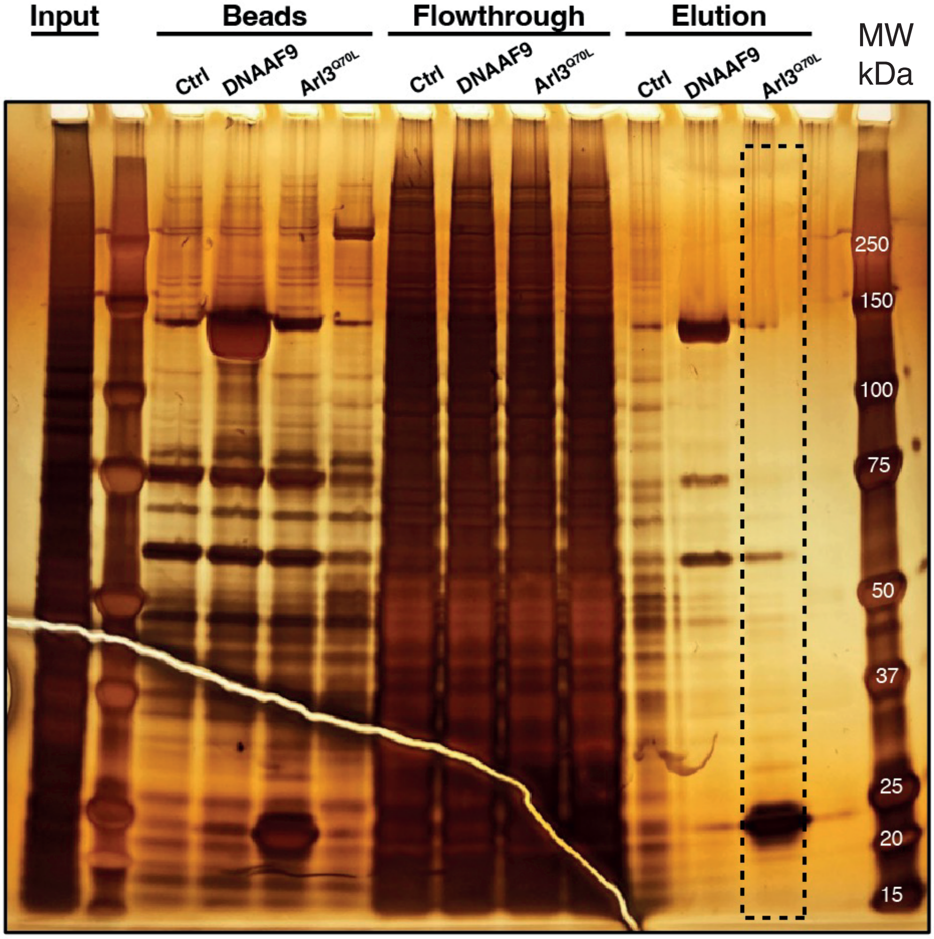

Regions shown in figures 3b and 3d are indicated by a dashed box

Gel and western blot source data, Figure 4

Figure 4e, left

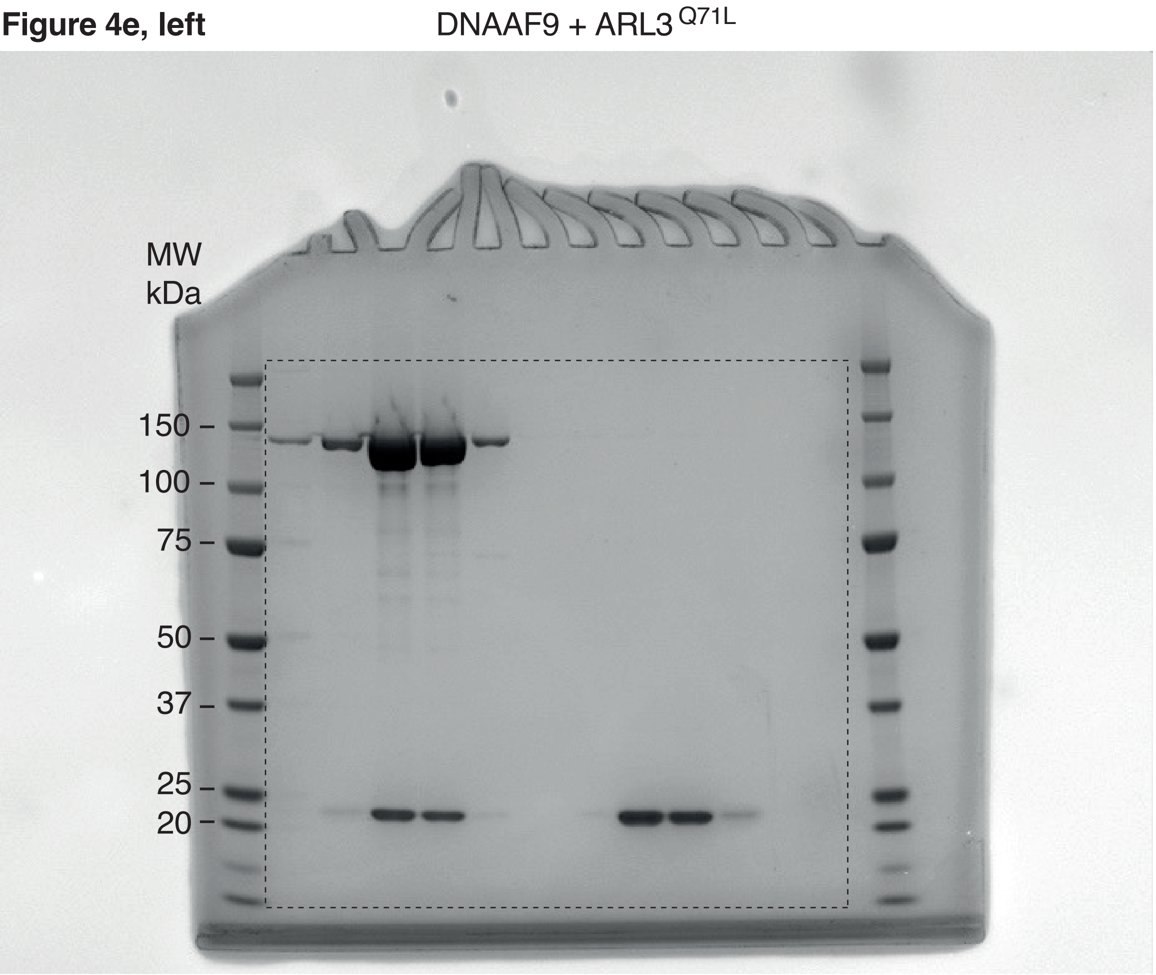

Figure 4e, right

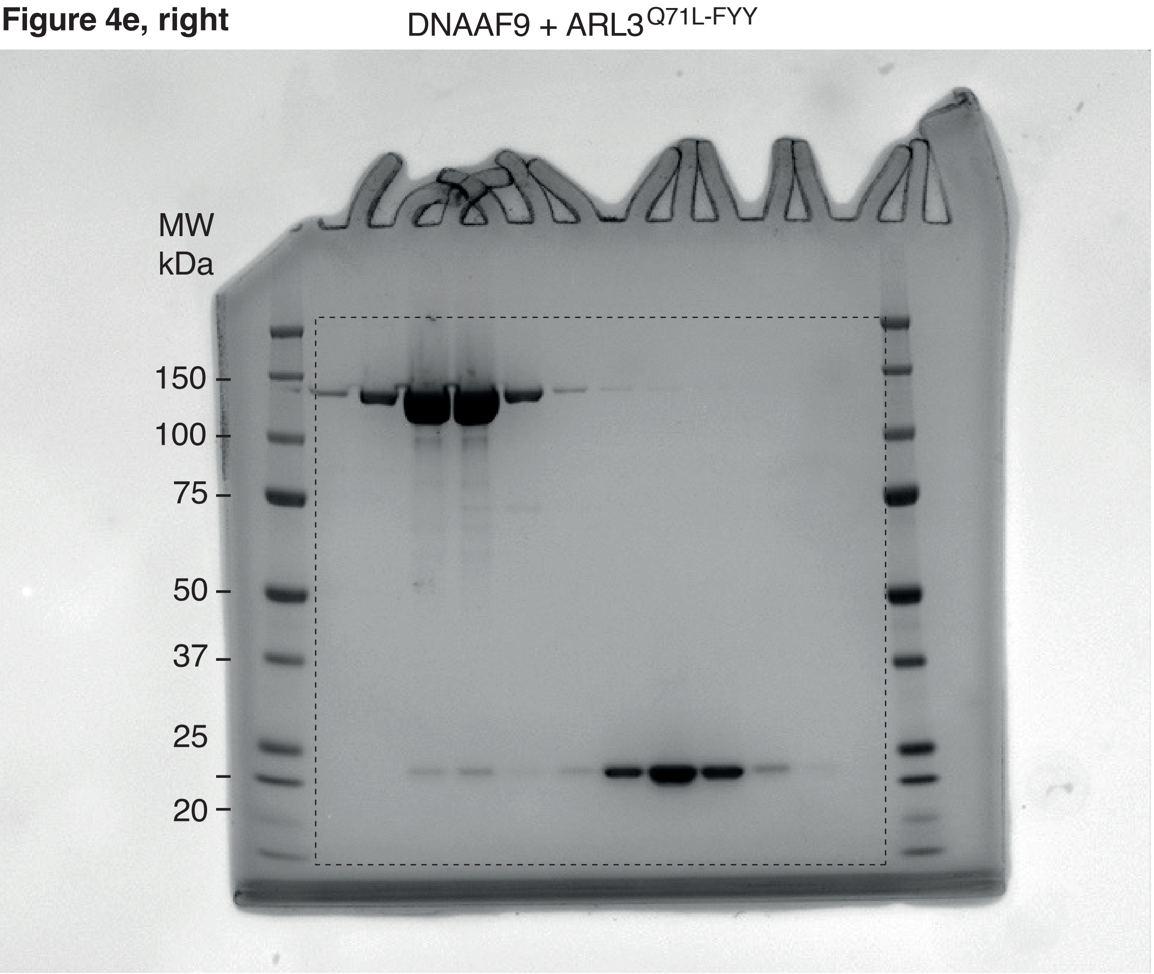

Regions shown in figure 4e are indicated by dashed boxes

Gel and western blot source data, Figure 5

Figure 5a, d

ODA-Shulin reconstitution

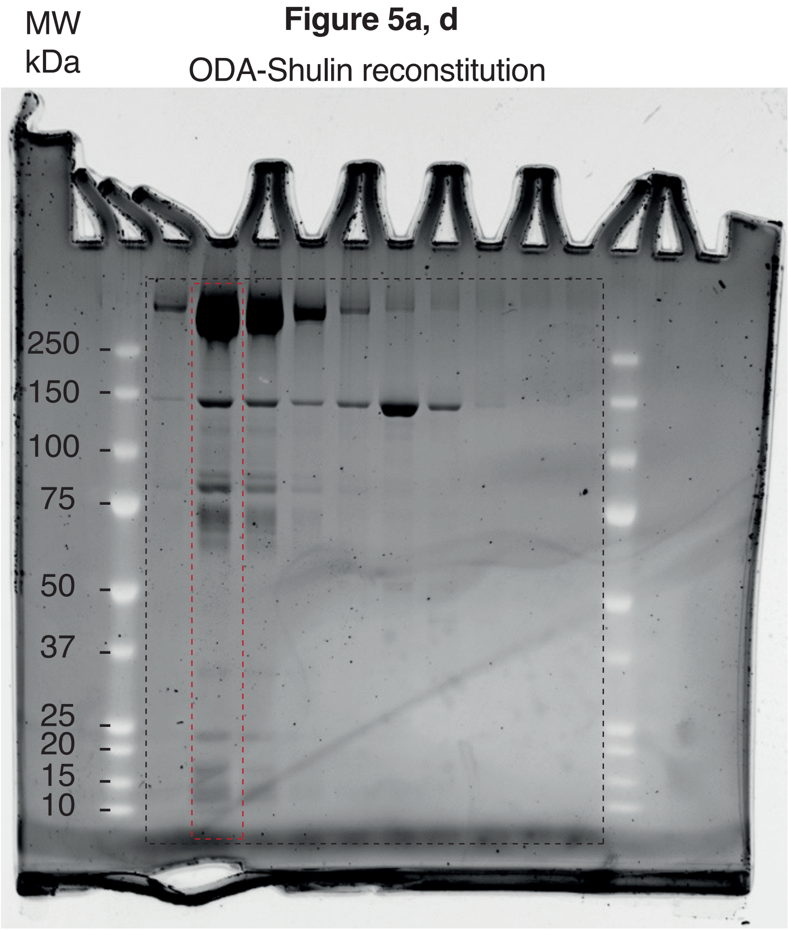

Figure 5b, d

Shulin displacement test with Arl3^{Q70L}

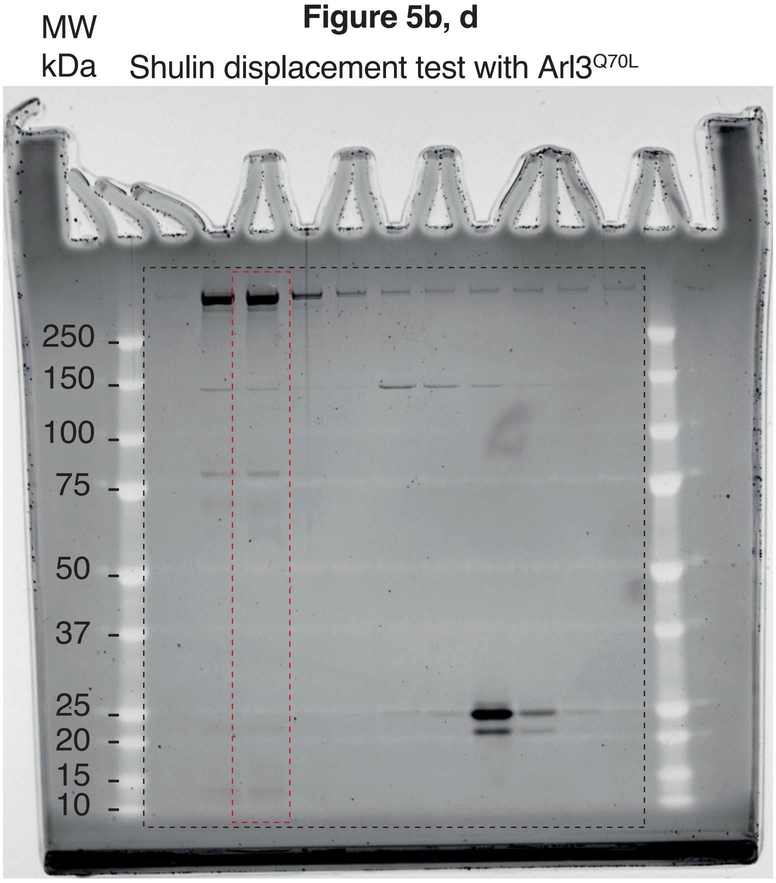

Figure 5c, d

Shulin displacement test with Arl3$^{Q70\text{-}FYY}$

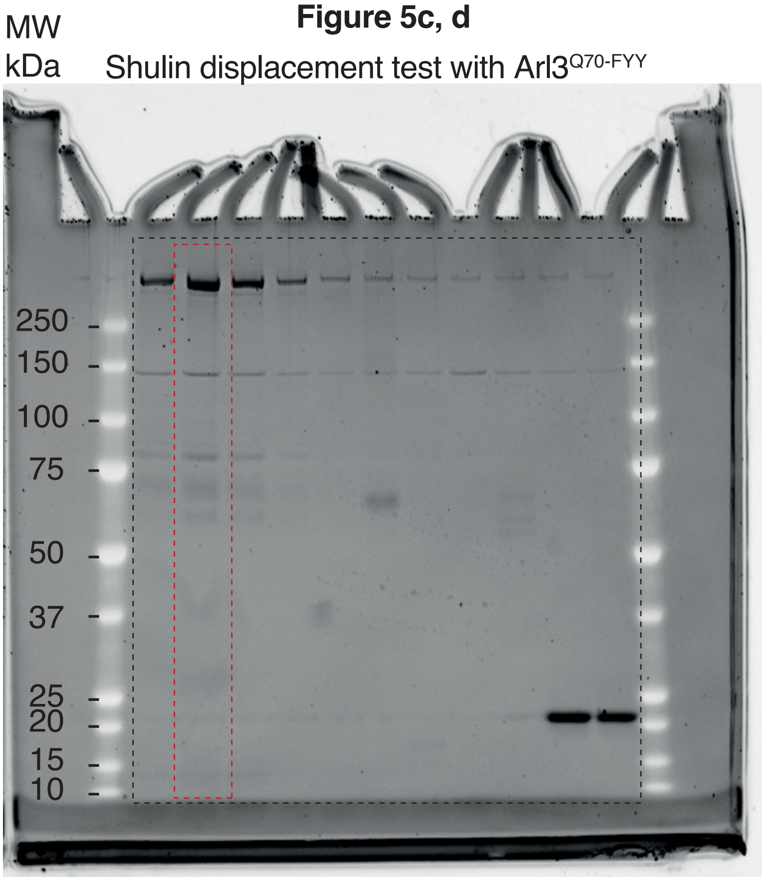

Regions shown in figures 5a, 5b and 5c are indicated by black dashed boxes

Regions shown in figure 5d are indicated by red dashed boxes

Gel and western blot source data, Extended Data Figure 4

Extended Data Figure 4a

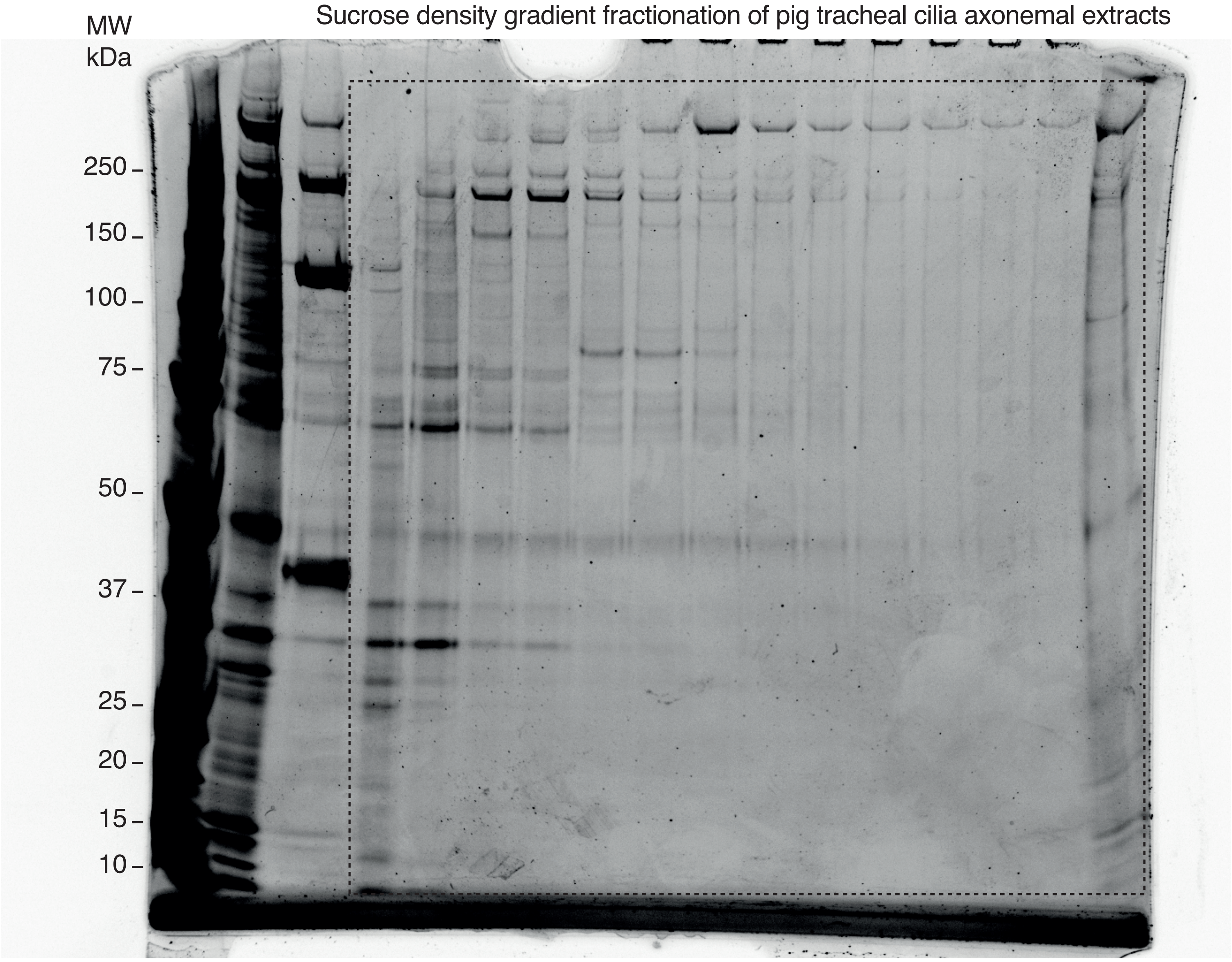

Region shown in extended data figure 4a is indicated by a dashed box

Gel and western blot source data, Extended Data Figure 7

Extended Data Figure 7c

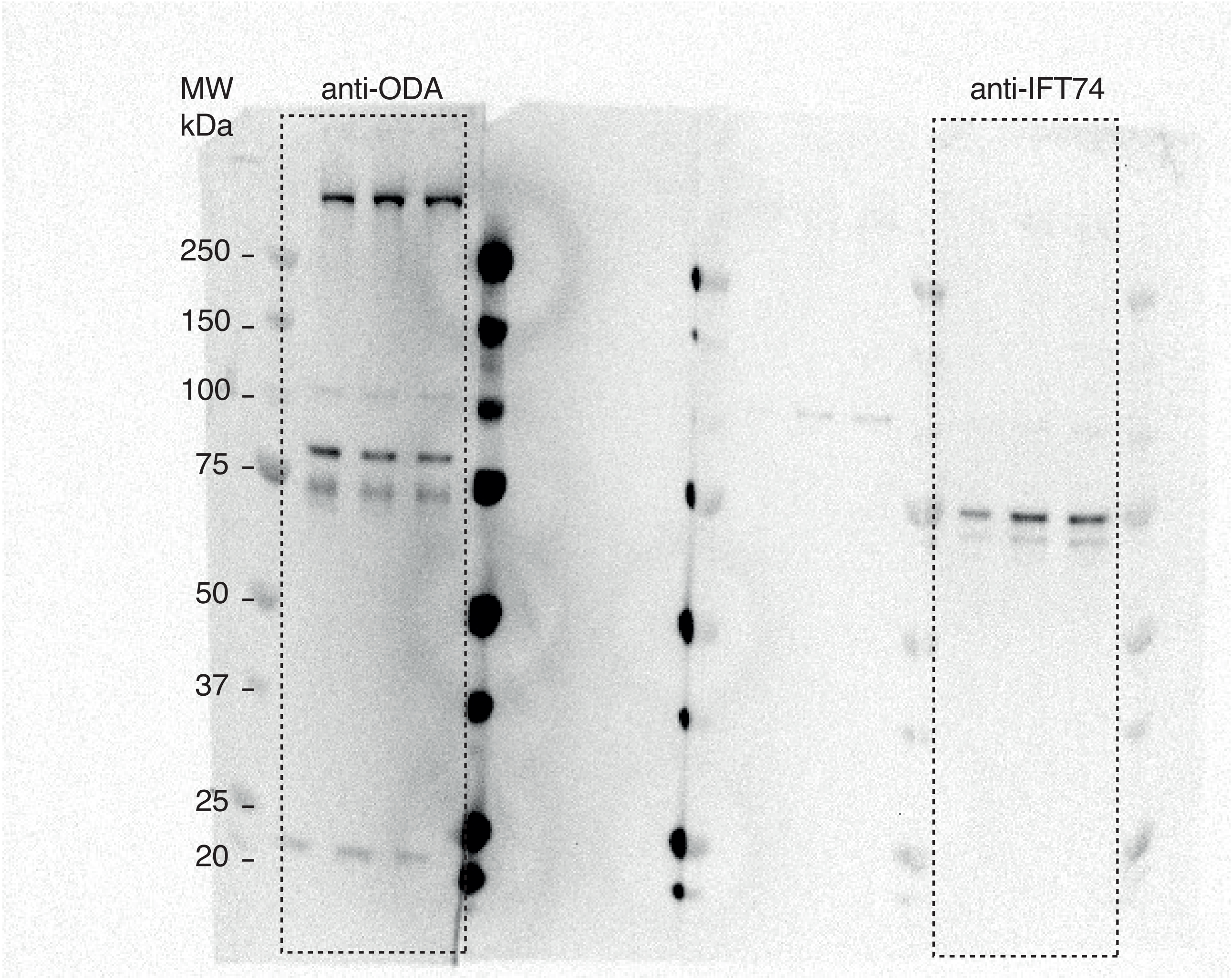

Longer exposure for middle two membranes

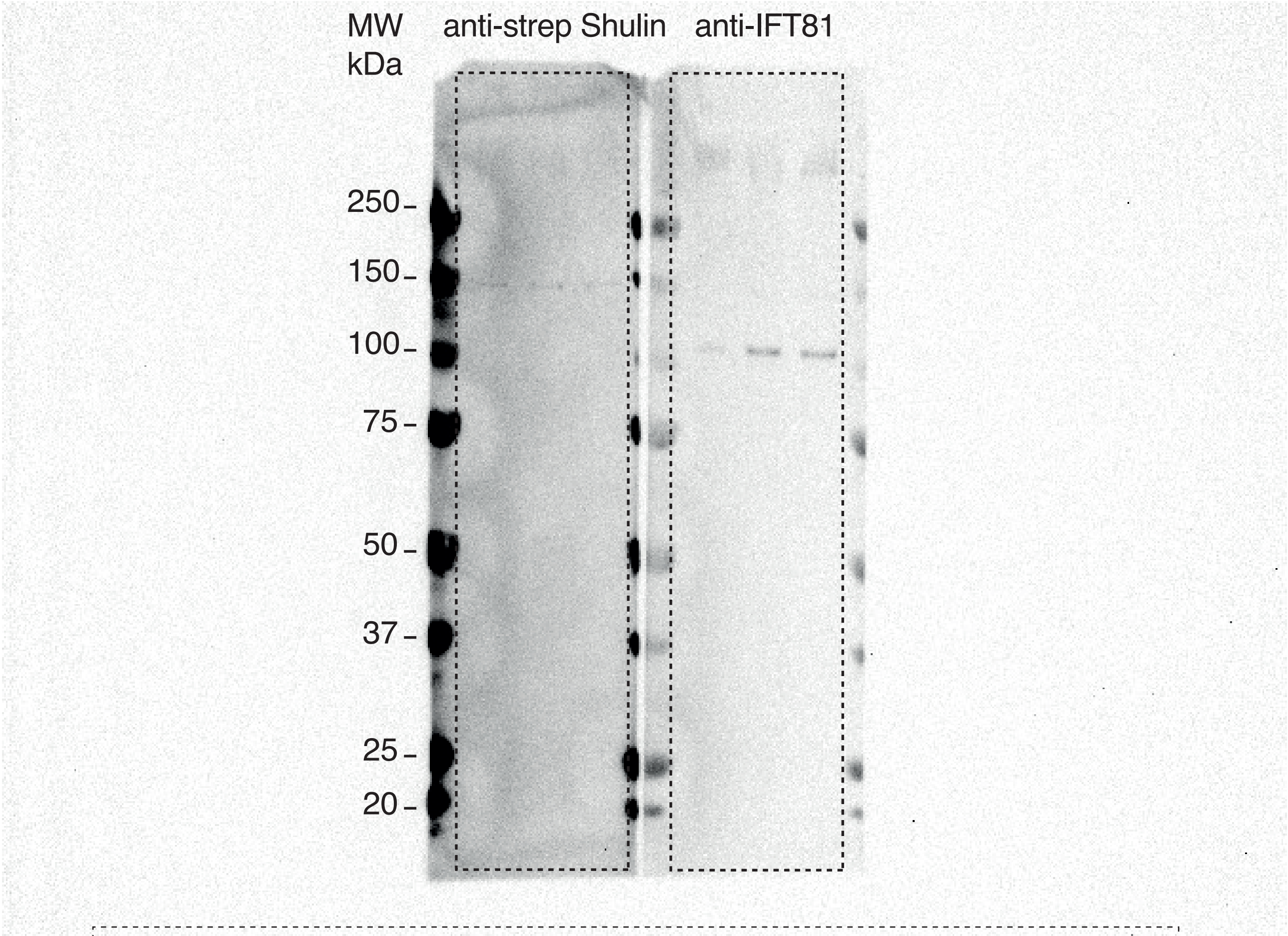

Region shown in extended data figure 7c are indicated by dashed boxes

Gel and western blot source data, Extended Data Figure 10

Extended Data Figures 10a-l

Regions shown in extended data figures 10a-l are indicated by dashed boxes

a DNAAF9 + ARL3^{Q71L}

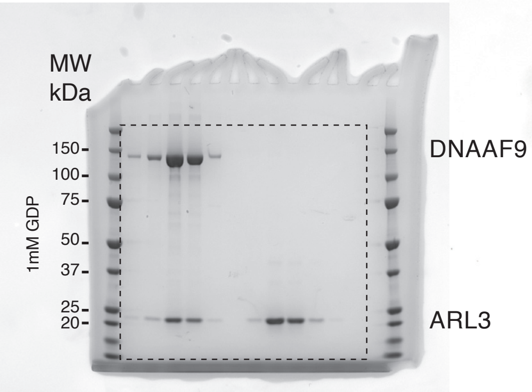

b DNAAF9 + ARL3^{T31N}

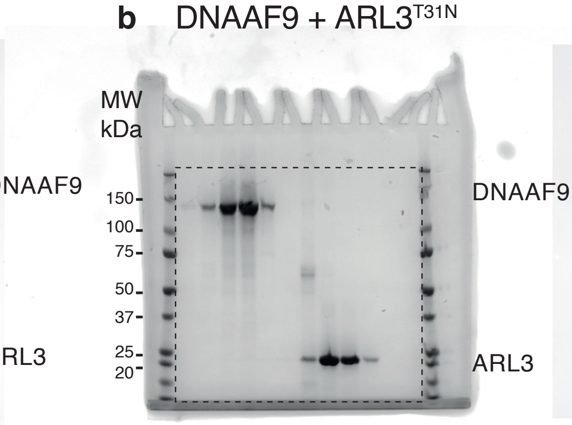

c DNAAF9 + ARL3$^{Q71L\text{-}FYY}$

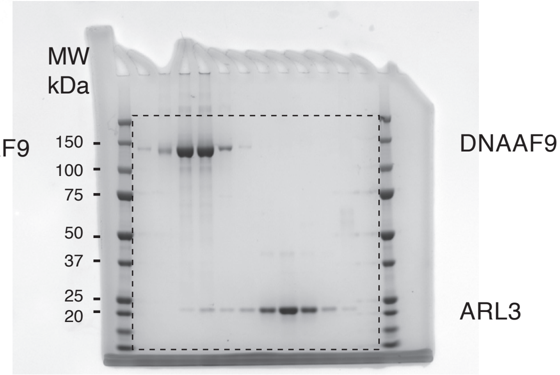

d

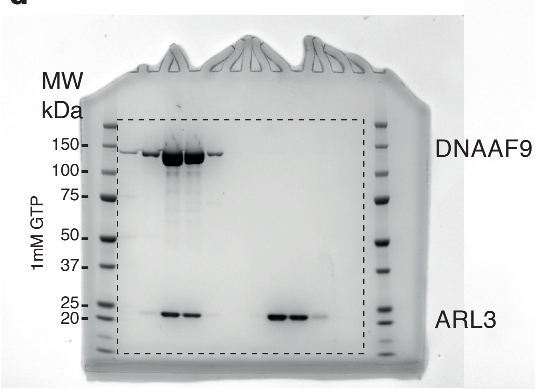

e

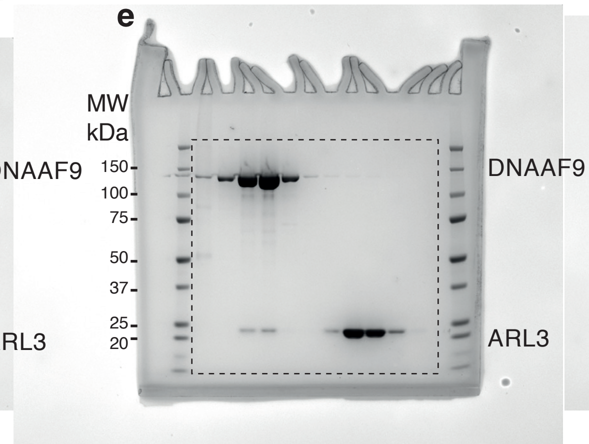

f

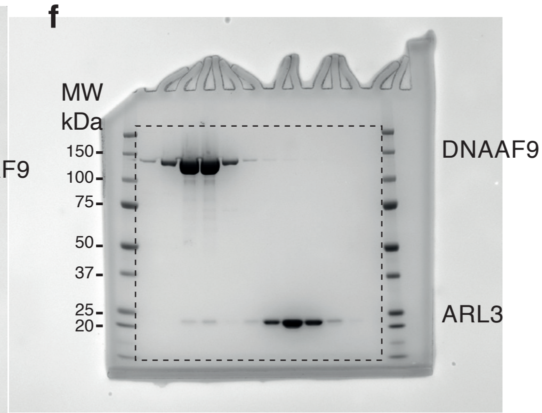

g Shulin + Arl3^{Q70L}

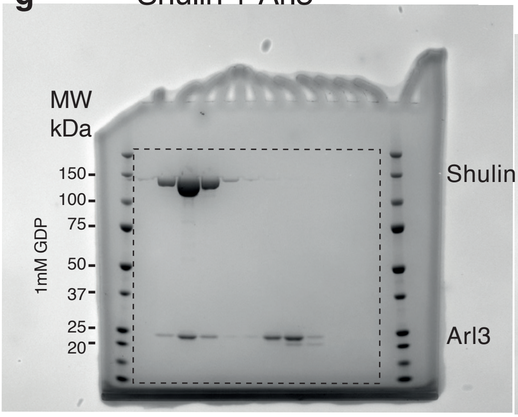

h Shulin + Arl3^{T30N}

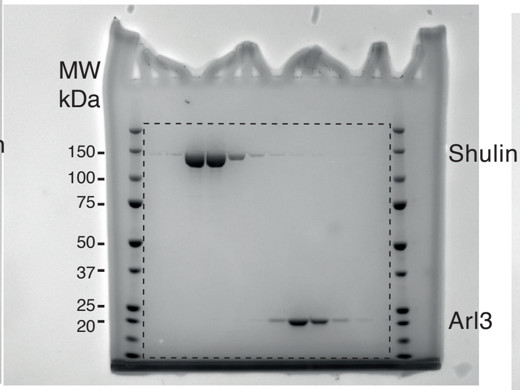

i Shulin + Arl3$^{Q70L\text{-}FYY}$

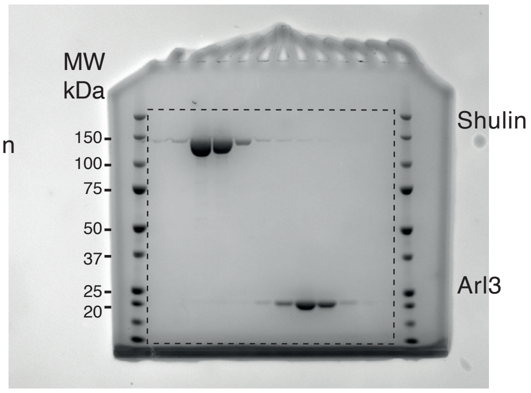

j

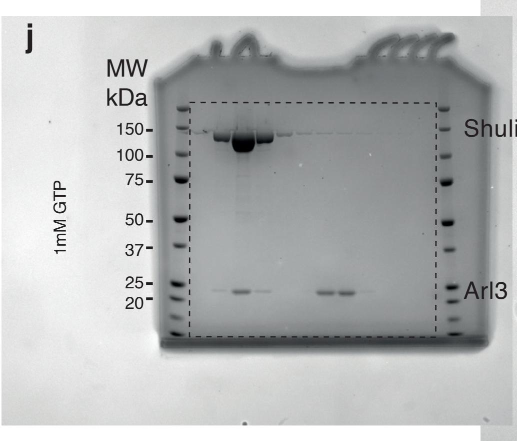

k

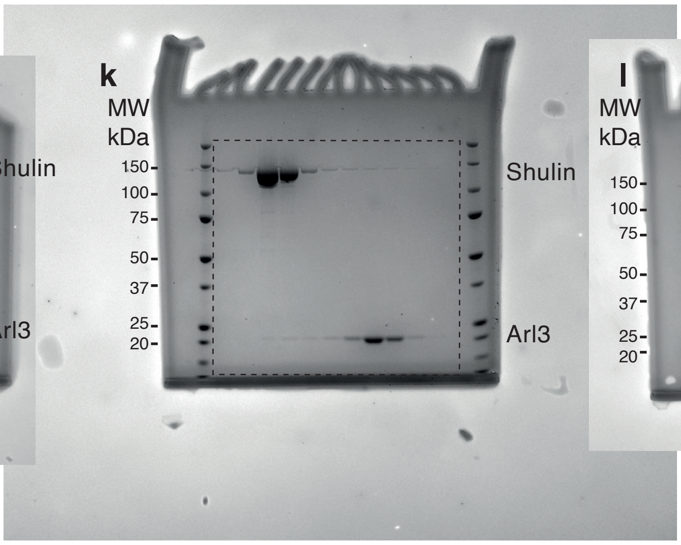

l

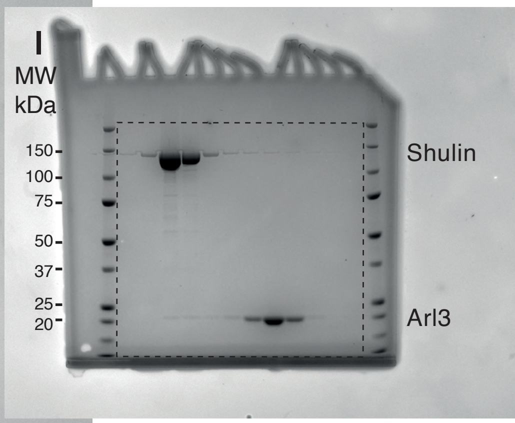

Supplement: Supplementary file 6 — Uncropped gels and western blots. [file 41594_2025_1680_MOESM6_ESM.pdf]
